# Patients with unilateral retinal vein occlusion show reduced radial peripapillary capillary density in their fellow eyes

**DOI:** 10.1186/s12886-021-02192-y

**Published:** 2021-12-27

**Authors:** Lingling Fan, Yazhou Zhu, Xiaomei Sun, Jinguo Yu, Hua Yan

**Affiliations:** 1 Department of Ophthalmology, The First Affiliated Hospital of Anhui Medical University, Hefei, Anhui China; 2 Department of Ophthalmology, Tianjin Medical University General Hospital, No. 154, Anshan Road, Tianjin, 300052 China

**Keywords:** Retinal vein occlusion, Optical coherent tomography angiography, Radial peripapillary capillary, Optic nerve head, Retinal nerve fiber layer, Vessel density

## Abstract

**Background:**

To evaluate the radial peripapillary capillary (RPC) density in the fellow eyes of unilateral retinal vein occlusion (RVO) patients using optical coherence tomography angiography (OCTA), and further analyze the correlation between RPC density and peripapillary retinal nerve fiber layer (RNFL) thickness.

**Methods:**

Seventy-eight unilateral RVO patients and 70 normal controls were included in the study. OCTA was conducted with the 4.5 × 4.5-mm scan pattern centered on the optic nerve head, and the RPC density and peripapillary RNFL thickness were quantified.

**Results:**

The peripapillary RNFL in the RVO fellow eyes was significantly thinner than in normal controls in the average, inferior-hemisphere, inferior quadrant, and temporal quadrant (*P* < 0.05, respectively). The RPC density in the fellow eyes was also significantly lower in the average, inferior-hemisphere, nasal quadrant, and temporal quadrant ((*P* < 0.05, respectively). There were no significant differences in RNFL thickness and RPC density between branch RVO fellow eyes and central RVO fellow eyes. Pearson’s correlation analysis showed significant positive correlations between the RPC density and RNFL thickness in all measurements (*P* < 0.001, respectively).

**Conclusions:**

The regional RPC density was reduced in the RVO fellow eyes, which might contribute to peripapillary RNFL thinning in the corresponding region, suggesting the influence of systemic risk factors on RVO. OCTA may offer new insights into the pathophysiology of RVO.

## Introduction

Retinal vein occlusion (RVO) is a relatively common retinal vascular disease and may cause significant loss of vision. RVO is subdivided into central RVO (CRVO) and branch RVO (BRVO) according to the blocked vessel. The pathogenesis of RVO is unclear and has been thought to be caused by vascular wall pathology, hemodynamic abnormalities, hemorheological changes, or compression of neighboring tissues, leading to thrombosis [1, 2].

Many studies have reported that the incidence of glaucoma in RVO patients is significantly higher than that in the general population, and, conversely, that the incidence of RVO in glaucoma patients is also higher than in the general population, suggesting a common pathogenic mechanism [3–6]. It has also been reported that the thickness of the retinal nerve fiber layer (RNFL) was reduced in the fellow eyes of unilateral RVO patients compared with that of normal control eyes [7]. Scripsema et al. reported a correlation between RNFL thinning and the decrease of peripapillary capillary density in glaucoma patients, suggesting that the pathophysiology of glaucoma is related to perfusion of the optic nerve [8]. However, it remains unclear whether the radial peripapillary capillary (RPC) network around the optic nerve head (ONH) in the fellow eyes of unilateral RVO patients is associated with peripapillary RNFL thinning.

Optical coherence tomography angiography (OCTA) is a non-invasive method of fundus imaging that provides high-resolution depth-resolved images of the retinal layers and choroid, and quantification of vascular densities and flow. It has been used in the clinical diagnosis and treatment of multiple ocular fundus diseases, including diabetic retinopathy (DR) [9, 10], age-related macular degeneration (AMD) [11] and pathological myopia [12, 13]. Several studies have reported decreased macular vessel densities in the deep capillary plexus together with enlargement of the foveal avascular zone in both RVO and fellow eyes using OCTA [14–16]. The present study aims to investigate the peripapillary RNFL thickness and RPC density using OCTA in the fellow eyes of patients with unilateral RVO, and to analyze their relationship.

## Methods

### Study and subjects

This observational retrospective study was conducted in accordance with the Declaration of Helsinki. The study was approved by the Ethics Committee of The First Hospital Affiliated of Anhui Medical University. The data was retrieved from the medical records of patients with unilateral RVO who attended the First Hospital Affiliated of Anhui Medical University from December 2019 to August 2020. The diagnosis of RVO was confirmed by fundus examination, aided by fundus photography. Some patients also underwent fundus fluorescein angiography (FFA). All treatment-naive unilateral RVO patients were included whether the RVO eyes were combined with macular edema or not. Patients were excluded if they were not treatment-naive or had a history of ocular surgery, glaucoma, optic nerve diseases, or retinal diseases including DR and AMD. The fellow eyes of the unilateral RVO patients were enrolled if the examination excluded ocular disease, apart from cataract, or if there was no previous history of ocular disease.

The control group comprised patients attending our outpatient department for health screening and were age-matched to the RVO patients. Controls had no history of ocular disease and showed no evidence of ocular disease, apart from cataracts, on ophthalmological examination. One of the two eyes was randomly selected.

All patients received detailed ophthalmologic examinations, including best corrected visual acuity (BCVA), slit lamp biomicroscopy, indirect ophthalmoscopy, fundus photography, and OCTA (RTVue-XR Avanti, Optovue, Inc., Fremont, CA). BCVA was measured by the international standard logarithmic visual acuity chart and converted to logMAR visual acuity for statistical analysis.

### Optical coherence tomography angiography

**Fig. 1:**
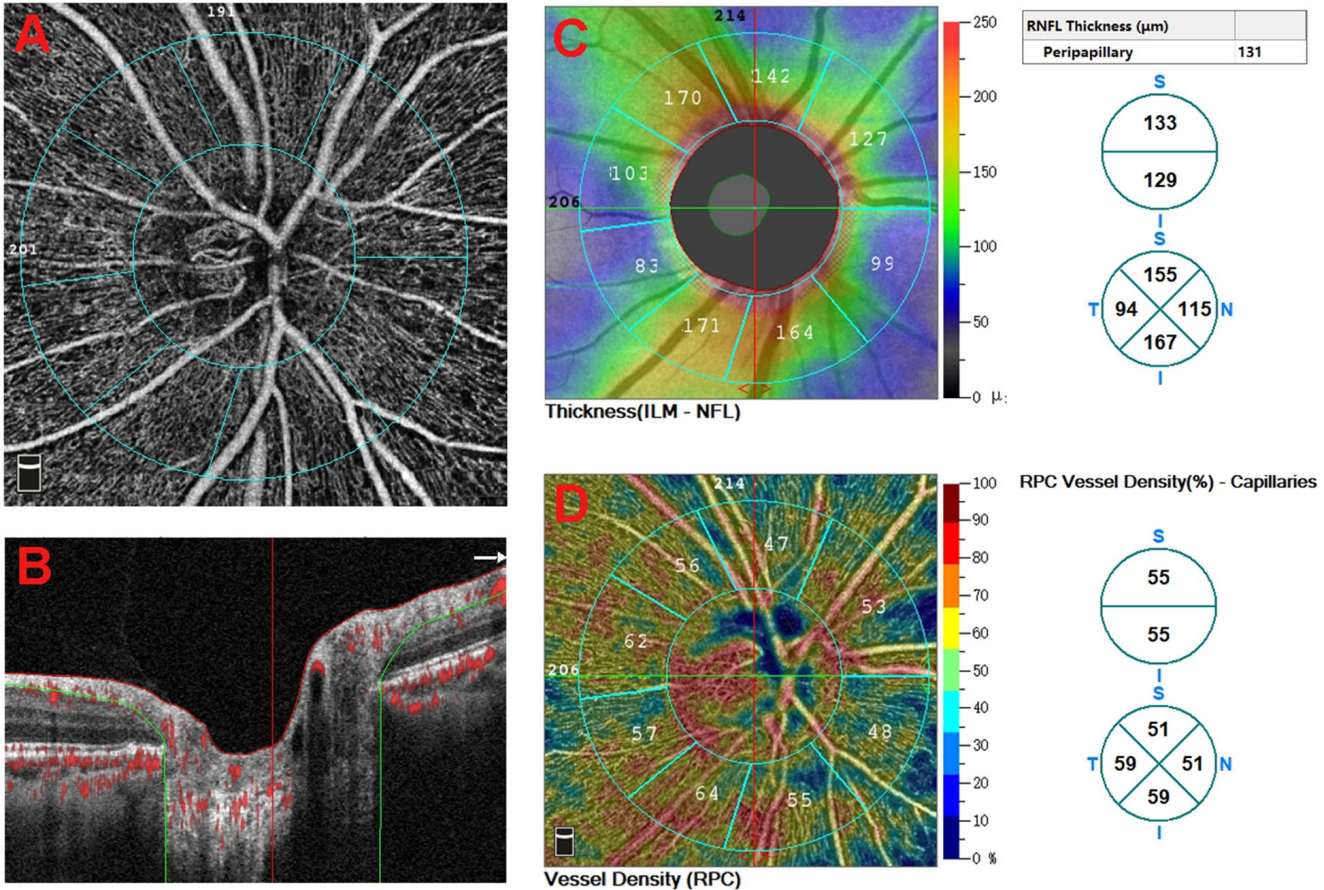
The images of OCTA 4.5 × 4.5-mm scan centered on the ONH. **A** The width of the outer ring was 1 mm, and the section was defined as peripapillary area. **B** The RPC density was analyzed in the superficial retinal layer from the RPC segment, which extends from the inner limiting membrane to the RNFL. **C** Color map of peripapillary RNFL thickness. **D** Color map of RPC density

OCTA imaging was performed with the RTVue XR Avanti device, and associated parameters were obtained automatically using the built-in AngioAnalytic software (version 2017.1.0.151; Optovue, Inc., Fremont, CA). The 4.5 × 4.5-mm scan pattern centered on the ONH was conducted in all eyes. The width of the outer ring was 1 mm, and the section was defined as peripapillary area (Fig. 1A). The vessel density of the peripapillary area was measured in the RPC segment superficial retinal layer (Fig. 1B). The thickness of the RNFL in the peripapillary area was also recorded. The color maps of RNFL thickness and RPC density are shown in Fig. 1C and D.

All the images were reviewed independently by two experienced fundus specialists (Jinguo Yu and Hua Yan). Poor images in which the scan quality index was below 5 and in which there were significant blink and motion artifacts were excluded. If automatic segmentation errors occurred, we corrected them manually.

### Statistical analysis

All data were expressed as means ± SD. The chi-square test was used to compare the sex and number of participants with hypertension in both groups. Student’s *t*-test for independent samples was used to compare the age, BCVA, RNFL thickness, and RPC density between each group. Pearson’s correlation analysis was used for analyzing the relationship between RNFL thickness and RPC density. *P* < 0.05 was considered statistically significant.

## Results

### Patient characteristics

A total of 78 patients with unilateral RVO were enrolled, and 70 participants were included in the normal control group. The characteristics of the participants in both groups are shown in Table 1. There were no significant differences in the sex, age, and presence of hypertension between the two groups (*P* = 0.56, 0.43 and 0.07, respectively). Additionally, there was no significant difference in BCVA between the fellow eyes of RVO patients and normal controls (*P* = 0.31).

**Table 1:** Characteristics of patients with unilateral RVO and normal controls

Characteristics	RVO	Control	***P***
**Sex (male/female)**	45/33	37/33	0.555
**Age (years)**	55.17 ± 9.32	54.04 ± 7.65	0.427
**Hypertension (n)**	19	9	0.07
**BCVA (LogMAR)**	−0.0323 ± 0.068	−0.022 ± 0.054	0.309

### Peripapillary retinal nerve fiber layer thickness

The peripapillary RNFL thicknesses of the fellow eyes in the RVO group and normal control eyes are listed in Table 2. The average RNFL thicknesses in the RVO fellow eyes were significantly thinner than those of controls (*P* = 0.02). Hemisphere measurement showed that the fellow eyes had thinner RNFL than controls in inferior-hemisphere measurement (*P* = 0.002). A similar result was found in the quadrant measurement where the RNFL of RVO fellow eyes was significantly reduced compared to controls in both the inferior and temporal quadrants (*P =* 0.04 and 0.01, respectively).

**Table 2:** Comparison of the peripapillary RNFL thickness between RVO fellow eyes and normal control eyes

RNFL thickness (μm)	RVO fellow eyes	Control eyes	***P***
**Average**	111.69 ± 10.01	115.54 ± 9.64	0.019
**Superior-hemisphere**	112.65 ± 10.36	114.76 ± 10.62	0.225
**Inferior-hemisphere**	110.51 ± 11.67	116.49 ± 10.9	0.002
**Superior**	138.38 ± 17.93	137.01 ± 14.68	0.614
**Inferior**	143.6 ± 18.23	149.71 ± 17.52	0.04
**Nasal**	95.23 ± 13.82	99.36 ± 12.79	0.062
**Temporal**	75.13 ± 12.35	80.3 ± 10.66	0.007

### Radial peripapillary capillary density

The RPC densities of the fellow eyes in the RVO group compared to the controls are shown in Table 3. The average vessel density of the fellow eyes was significantly lower than that of the controls (*P* = 0.03). In the hemisphere measurement, the RVO fellow eyes showed significantly lower vessel densities than controls in the inferior-hemisphere measurement (*P =* 0.02) and the same trend in the quadrant measurement in both the nasal and temporal quadrants (*P =* 0.03 and 0.02, respectively).

**Table 3:** Comparison of the RPC density between RVO fellow eyes and normal control eyes

RPC density (%)	RVO fellow eyes	Control eyes	***P***
**Average**	52.45 ± 2.74	53.44 ± 2.6	0.026
**Superior-hemisphere**	52.73 ± 2.97	53.43 ± 3.02	0.16
**Inferior-hemisphere**	52.28 ± 2.92	53.36 ± 2.5	0.017
**Superior**	54.03 ± 4.44	53.8 ± 3.34	0.718
**Inferior**	55.26 ± 3.48	55.77 ± 3.6	0.384
**Nasal**	48.35 ± 3.79	49.67 ± 3.39	0.028
**Temporal**	53.78 ± 4.68	55.41 ± 3.79	0.022

### OCTA parameters in the fellow eyes of BRVO and CRVO, and control eyes

The fellow eyes of the RVO patients were divided into two subgroups, BRVO fellow eyes (*n* = 46) and CRVO fellow eyes (*n* = 32). The OCTA parameters including peripapillary RNFL thickness and RPC density are shown in Table 4, and they were compared with normal controls, respectively. The RNFL thicknesses were significantly thinner than controls in BRVO fellow eyes in average, inferior-hemisphere, nasal quadrant and temporal quadrant measurements (*P* = 0.039, 0.007, 0.03 and 0.006, respectively), and in CRVO fellow eyes in inferior-hemisphere (*P* = 0.013). The RPC densities were significantly lower than controls in BRVO fellow eyes in nasal quadrant (*P* = 0.019), and in CRVO fellow eyes in average, superior-hemisphere, inferior-hemisphere and temporal quadrant measurements (*P* = 0.014, 0.03, 0.027 and 0.002, respectively). However, there were no significant differences between the BRVO fellow eyes and CRVO fellow eyes.

**Table 4:** OCTA parameters in the BRVO fellow eyes, CRVO fellow eyes and control eyes

Measurements	RNFL thickness (μm)						RPC density (%)					
	Control eyes	BRVO fellow eyes	CRVO fellow eyes	***P1***	***P2***	***P3***	Control eyes	BRVO fellow eyes	CRVO fellow eyes	***P1***	***P2***	***P3***
**Average**	115.54 ± 9.64	111.59 ± 10.47	111.84 ± 9.48	0.039	0.074	0.912	53.44 ± 2.6	52.73 ± 2.76	52.04 ± 2.71	0.167	0.014	0.272
**Superior-hemisphere**	114.76 ± 10.62	112.67 ± 10.64	112.63 ± 10.11	0.304	0.342	0.984	53.43 ± 3.02	53.23 ± 2.91	52.03 ± 2.97	0.719	0.03	0.079
**Inferior-hemisphere**	116.49 ± 10.9	110.41 ± 12.56	110.66 ± 10.44	0.007	0.013	0.929	53.36 ± 2.5	52.38 ± 3.11	52.13 ± 2.68	0.062	0.027	0.722
**Superior**	137.01 ± 14.68	139.8 ± 17.49	136.34 ± 18.62	0.356	0.845	0.405	53.8 ± 3.34	54.55 ± 4.56	53.28 ± 4.21	0.338	0.505	0.215
**Inferior**	149.71 ± 17.52	143.7 ± 19.6	143.47 ± 16.36	0.087	0.091	0.957	55.78 ± 3.6	55.27 ± 3.55	55.25 ± 3.45	0.463	0.493	0.979
**Nasal**	99.36 ± 12.79	93.74 ± 14.37	93.38 ± 14.37	0.03	0.471	0.256	49.67 ± 3.39	48.03 ± 3.95	48.81 ± 3.55	0.019	0.245	0.375
**Temporal**	80.3 ± 10.66	75.02 ± 8.48	74.97 ± 16.71	0.006	0.105	0.987	55.41 ± 3.79	54.41 ± 3.58	52.41 ± 5.45	0.157	0.002	0.053

### Correlation between peripapillary RNFL thickness and RPC density

**Fig. 2:**
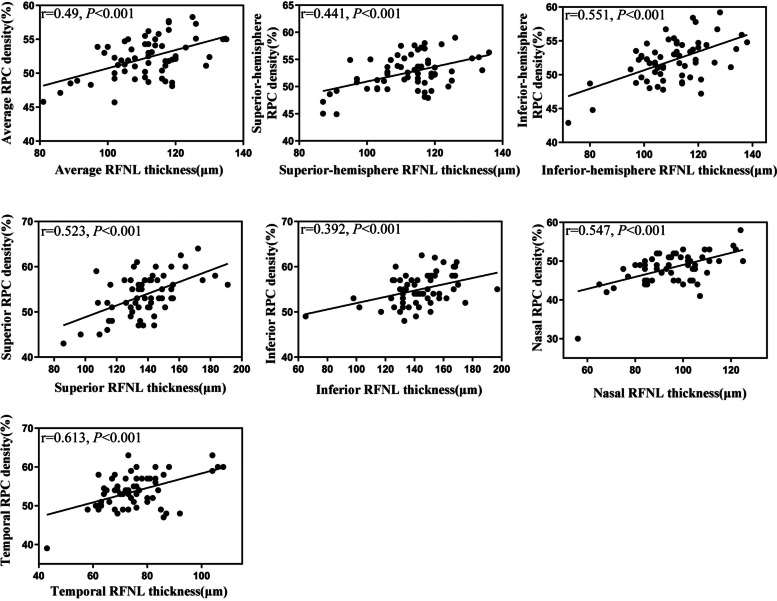
Scatter plots showing the linear correlation of RPC density and peripapillary RNFL thickness in average, hemisphere, and quadrant measurements, respectively

The results of the correlation analyses between peripapillary RNFL thickness and RPC density in the average, hemisphere, and quadrant measurements are shown in Fig. 2. The average peripapillary RNFL thickness and RPC density showed a significant positive correlation (*r* = 0.49, *P* < 0.001). Strong correlations were also found in the hemisphere measurements in both the superior and inferior hemisphere (*r* = 0.44, *P* < 0.001; *r* = 0.55, *P* < 0.001, respectively). In the quadrant measurement, peripapillary RNFL thickness showed significant positive correlations with RPC density in all four quadrants (superior quadrant *r* = 0.52, *P* < 0.001; inferior quadrant *r* = 0.39, *P* < 0.001; nasal quadrant *r* = 0.55, *P* < 0.001; temporal quadrant *r* = 0.61, *P* < 0.001).

## Discussion

In the study, we found that in the fellow eyes of unilateral RVO patients, there was a significant decrease in the peripapillary RNFL thickness in the average, inferior-hemisphere, inferior quadrant, and temporal quadrant, which is consistent with the previous study [7] and the change is similar to that seen in glaucoma. Lopilly Park et al. reported that glaucoma in the fellow eyes of patients with unilateral BRVO progressed more rapidly compared with glaucoma patients without RVO [17], suggesting the presence of a similar vascular abnormality in both diseases [18–20]. In the present study, therefore, we further evaluated the RPC network in RVO patients’ fellow eyes using OCTA and analyzed whether it was associated with RNFL thinning.

OCTA is a novel technique that combines en face OCT reconstruction with motion contrast processing to show the vasculature of the retina and choroid in various layers. The movement of erythrocytes within the blood vessels is detected with the SSADA algorithm, generating images of the perfused vessels in a rapid, noninvasive, and repeatable manner [21]. A limitation of OCTA, however, is an inability to provide information on dynamic blood flow and vascular leakage information, as is provided by conventional FFA. Currently, both macular and ONH capillaries can be visualized using the SSADA algorithm. There are many studies analyzing the status of vascular perfusion in macular and ONH in various conditions via OCTA [10, 22, 23].

Microvascular changes are visible in both deep and superficial capillary networks of the retina in RVO eyes using OCTA, such as non-perfusion areas, vascular tortuosity, capillary engorgement, and formation of collateral vessels [14, 15, 24]. Some groups also have assessed macular microvascular changes in the fellow eyes of unilateral RVO patients via OCTA [15, 16], and the results demonstrated the vessel densities in both deep and superficial capillary plexus were decreased, especially the deep capillary plexus. Isolated deep retinal capillary ischaemia was also found in both RVO eyes and their fellow eyes [25]. The status of choriocapillaris in RVO fellow eyes has also been studied via OCTA, and the results showed a substantial decrease of perfusion in choriocapillaris [26, 27]. However, the retinal microvasculature in the ONH of RVO patients has rarely been studied via OCTA. To the best of our knowledge, there are only two studies. Shin et al. used the Cirrus HD-OCT 5000 with AngioPlex device to evaluate peripapillary microvascular parameters, and they found that the peripapillary vessel density and perfusion density as well as RNFL thickness were decreased in RVO fellow eyes [28]. In addition to this, Ozcaliskan et al. also used the Cirrus HD-OCT 5000 with AngioPlex device to assess macular microvasculature and RPC plexus in RVO fellow eyes, and they concluded that the presence of RVO might be related to retinal microvasculature changes in both the macular and peripapillary regions of the fellow eyes [29]. In our study, RPC density was evaluated using a different OCTA devices (AngioVue RTVue XR Avanti), and consistent with the two studies, we found lower RPC densities in the fellow eyes than in normal controls in the average, inferior-hemisphere, nasal quadrant, and temporal quadrant measurements, similar to the decreases observed in RNFL thinning. Combined with the previous researches and our study, we concluded that both retinal and choroidal microvascular changes in RVO eyes as well as their fellow eyes, and the alteration may appear before RVO occurs. Various systemic diseases are considered as risk factors of RVO, including hypertension, diabetes, hyperlipidemia, and arteriosclerosis [1, 2]. The study results further conform the link between systemic risk factors and RVO, which can simultaneously affect both eyes.

However, the study of Adhi et al. showed decreased macular vessel perfusion of deep retinal plexus and vessel tortuosity were observed more frequently in the CRVO fellow eyes than BRVO fellow eyes, and the FAZ area was larger in CRVO fellow eyes than BRVO fellow eyes [15]. We are confused with these results, which may demonstrate the existence of local factors. Thus, subgroup analysis was done further, and the results showed no significant differences in all parameters between the BRVO fellow eyes and CRVO fellow eyes. Additionally, the OCTA parameters of BRVO fellow eyes and CRVO fellow eyes were also compared with control eyes, respectively, and the change trends were similar to that of whole RVO fellow eyes. This may demonstrate the possibility of local factors affecting the retinal microcirculation in RVO fellow eyes is very small, and the study results of Adhi et al. maybe due to the different severity of systemic risk factors in patients with BRVO and CRVO. RPCs form a special capillary network, located within the RNFL and most prominent in the arcuate RNFL region. Histological and clinical examinations have shown that the RPC network may be, to some degree, responsible for RNFL nourishment around the ONH in normal eyes [30–32]. Additionally, the size of the RPC network has been shown to be correlated with RNFL in various conditions, including glaucoma, DR, and non-arteritic anterior ischemic optic neuropathy [10, 11, 28, 33, 34]. Thus, we further analyzed the association between RPC density and RNFL thickness using different measurements. The results showed significant positive correlations for all measurements, suggesting that the reduction in RPC density may contribute to peripapillary RNFL thinning in the fellow eyes of unilateral RVO patients. In other words, the changes of RPC microcirculation lead to structural changes in RVO fellow eyes.

Our study has some limitations. The RPC was detected at 8.5 mm from the ONH edge on the temporal side using OCTA [30]. Thus, the small scan window (4.5 × 4.5 mm) just allows analysis of a limited area of the RPC, without reflecting more peripheral changes, and a larger measurement area is better. Additionally, while elevated intraocular pressure (IOP) is an important contributor to RNFL thinning, it was not detected in every subject. Thus, the influence of IOP on RNFL thinning cannot be excluded, suggesting further investigation.

In conclusion, the present study demonstrated a reduction in the regional RPC density in the fellow eyes of unilateral RVO patients., which may contribute to the peripapillary RNFL thinning in the corresponding region. This may further confirm the effect of systemic risk factors on RVO, leading to changes of retinal microcirculation in both eyes of patients with unilateral RVO. The study also showed the potential for OCTA in the detection of previous occlusive disease or early pathological changes indicative of future occurrence. Future longitudinal studies with more patients are required to help understand the pathophysiology and natural progression of RVO and guide the clinical application of OCTA.

## Data Availability

The datasets used and analyzed during the present study are available from the corresponding author on reasonable request.

## Abbreviations

RVO: Retinal vein occlusion
CRVO: Central retinal vein occlusion
BRVO: Branch retinal vein occlusion
RNFL: Retinal nerve fiber layer
RPC: Radial peripapillary capillary
ONH: Optic nerve head
OCTA: Optical coherence tomography angiography
DR: Diabetic retinopathy
AMD: Age-related macular degeneration
FAZ: Foveal avascular zone
FFA: Fundus fluorescein angiography
BCVA: Best corrected visual acuity
